# Duhuo Jisheng Decoction regulates intracellular zinc homeostasis by enhancing autophagy via PTEN/Akt/mTOR pathway to improve knee cartilage degeneration

**DOI:** 10.1371/journal.pone.0290925

**Published:** 2024-01-02

**Authors:** Ye-Hui Wang, Yi Zhou, Xiang Gao, Sheng Sun, Yi-Zhou Xie, You-Peng Hu, Yang Fu, Xiao-Hong Fan, Quan Xie

**Affiliations:** 1 Hospital of Chengdu University of Traditional Chinese Medicine, Chengdu, Sichuan, China; 2 Sichuan Province Orthopedic Hospital, Chengdu, Sichuan, China; 3 Chongqing Traditional Chinese Medicine Hospital, Chongqing, China; University College London, UNITED KINGDOM

## Abstract

**Background:**

Articular cartilage and cartilage matrix degradation are key pathological changes occurring in the early stage of knee osteoarthritis (KOA). However, currently, there are limited strategies for early prevention and treatment of KOA. Duhuo Jisheng Decoction (DHJSD) is a formula quoted in *Bei Ji Qian jin Yao Fang*, which was compiled by Sun Simiao in the Tang Dynasty of China. As a complementary therapy, it is widely used to treat early-stage KOA in China; however, its mechanism has not been completely elucidated.

**Objective:**

This study investigated the potential role of DHJSD in preventing cartilage degradation and the underlying mechanism.

**Methods:**

A rat model of KOA model was established via the Hulth method. Subsequently, 25 rats were randomized into sham (saline), model control (saline), high-DHJSD (1.9g/mL of DHJSD), medium-DHJSD (1.2g/mL of DHJSD), and low-DHJSD groups (0.6g/mL of DHJSD). After 4 weeks of treatment, all rats were sacrificed and the severity of the cartilage degeneration was evaluated by a series of histological methods. The autophagosome was observed using transmission electron microscopy, and the related functional proteins were detected by the western blotting and real-time polymerase chain reaction. Next, the mechanism by which DHJSD improves knee cartilage degeneration was further clarified the in vitro by gene silencing technology combined with a series of functional experiments. The proteins levels of PTEN, Akt, p-Akt, mTOR, and p-mTOR, as well as the marker proteins of autophagy and apoptosis were determined. Zinc levels in chondrocytes were determined using inductively coupled plasma mass spectrometry.

**Results:**

Histopathological staining revealed that DHJSD had a protective effect on the cartilage. DHJSD increased autophagosome synthesis and the expression of autophagy proteins LC3 and Beclin-1 in chondrocytes. Moreover, it reduced the phosphorylation levels of Akt and mTOR and the levels of zinc, MMP-13, Bax, and Bcl-2. Following *PTEN* silencing, this DHJSD-mediated reduction in Akt and mTOR phosphorylation and Bax, Bcl-2, and zinc levels were further decreased; in addition, DHJSD-mediated increase in LC3 and Beclin-1 levels was decreased.

**Conclusion:**

DHJSD inhibits the Akt/mTOR signaling pathway by targeting *PTEN* to promote autophagy in chondrocytes, which may help reduce MMP-13 production by regulating zinc levels in chondrocytes.

## Introduction

Knee osteoarthritis (KOA) is a common clinical disease in orthopedic surgery, with a prevalence of 25.51% in China [1]. Particularly in China, with an increase in population aging and average life expectancy, the prevalence of KOA is estimated to rise [2]. Reportedly, KOA is caused by several factors including trauma, immune abnormalities, degeneration, and metabolic abnormalities. The late stage of KOA is characterized by irreversible bone hyperplasia, joint surface degeneration, and joint space narrowing. Conservative treatment is often ineffective, and surgery is almost the only option for the late stage of KOA [3]. At present, knee arthroplasty is the most effective surgical method to reduce knee pain and improve knee function. However, this surgery is associated with the risk of surgical complications, such as surgical failure, infection, and anesthesia risk; in addition the cost of surgical treatment is relatively high [4]. Therefore, early treatment of KOA is of great significance in disease management. The primary goals of the treatment of early KOA include delaying the degeneration of articular cartilage and relieving pain. Although opioids and non-steroidal anti-inflammatory drugs have definite effects in relieving pain, these are associated with a risk of gastrointestinal tract and cardiovascular adverse events [5]. Moreover, the long-term clinical efficacy of diacerein and glucosamine is controversial [6–8].

Duhuo Jisheng Decoction (DHJSD) is a formula quoted in *Bei Ji Qian jin Yao Fang*, a Chinese medicine compilation by Sun Simiao from the Tang Dynasty of China. As a complementary therapy, DHJSD is widely for the management of bone and joint diseases in China; however, its exact mechanism has not been completely elucidated. Existing studies have confirmed that DHJSD regulates inflammatory factors, reduces apoptosis and pyroptosis of nucleus pulposus cells, and inhibits extracellular matrix degradation [9–12], indicating the multiple mechanisms of action of DHJSD in osteoarthritis (OA).

The pathogenesis of OA involves the joint action of several factors, with articular cartilage degeneration being primarily mediated by excessive degradation of the extracellular matrix [13]. Notably, the degradation of chondrocytes matrix mediated by zinc is an important link in cartilage degeneration [14,15]. It has been reported that intracellular zinc homeostasis plays an important role in autophagy [16–18]. Therefore, autophagy may be closely associated with the regulation of zinc homeostasis in chondrocytes and may be an upstream mechanism of zinc-mediated cartilage degeneration. Studies have shown that DHJSD can reduce the level of intracellular zinc and interfere with the degradation of the cell matrix to induce a chondrocyte protective effect [19].

The PI3K/Akt/mTOR pathway is an important intracellular autophagy signal regulation pathway. Activation of this pathway can regulate cell growth and metabolism, inhibit activity of autophagy-related proteins, and reduce autophagy, which have implications for cell growth, energy metabolism, and pathogenesis of several diseases [20,21]. In particular, the PI3K/Akt/mTOR signaling pathway plays a critical role in autophagy among chondrocyte [21]. LC3 is a molecular marker of autophagy that is located in the cytoplasm. During autophagy, LC3-I binds covalently to phosphatidyleolamine to form LC3-II and localizes on the autophagosome membrane to participate in the formation of autophagosome [22]. Inhibition of this pathway has been reported to increase the level of autophagy [23]. In addition, phosphatase and tensin homolog deleted on chromosome ten (*PTEN*) is a typical tumor suppressor gene that inhibits the PI3K/Akt/mTOR growth signaling cascade [24]. This pathway can be regulated via multiple factors [25], and an alteration in these factors will lead to the dysregulation of this pathway and other pathways, leading to overgrowth [26]. The PTEN protein comprises four heterogeneous structures, PTEN-L, PTEN-M, PTEN-N and PTEN-O; of these, PTEN-L plays an important role in regulating the PI3K/Akt/mTOR signaling pathway as well as autophagy [27,28]. In addition, *PTEN* is a typical tumor suppressor gene that inhibits the PI3K/Akt/mTOR signaling cascade, thereby increasing autophagy and reducing apoptosis [29]. Accordingly, the present study evaluated the effect of DHJSD on cartilage and evaluated whether this effect was mediated via the PTEN/Akt/mTOR, autophagy, and zinc homeostasis in chondrocytes.

## Materials and methods

### Reagents

RiboFECT^TM^CP(R10035.8) and siRNA(R10043.8) were supplied by RiboBio(Guangzhou, China). IP cell lysate (P0013) and BCA Protein Concentration Assay Kit (P0009) were procured from Beyotime (Shanghai, China). Torchlight Hypersensitive ECL Western HRP Substrate (17046) was purchased from Zen-bioscience (Chengdu, China). Goat anti-rabbit immunoglobulin (Ig) G (H+L) HRP was purchased from Affinity (Jiangsu, China). Antibodies against β-actin (AC026), Bax (A19684), Bcl-2 (A20777), Beclin-1 (A7353), LC3 (A5618), mTOR (A11355), p-mTOR (AP0094), Akt (A18675), p-Akt (AP0980), PTEN (A19104), and HRP goat anti-mouse IgG (H+L) (AS003) were procured from ABclonal (Wuhan, China). Antibodies against collagen II (NB600-844) were obtained from NOVUS (Shanghai, China). The TRAP staining kit (CR2203125, Spec: 50T) and secondary antibodies against collagen II (GB23301) were purchased from ServiceBio (Wuhan, China). The DAB kit (ZLI-9018) was provided by ZSGB-BIO (Beijing, China).

DHJSD was formulated by combining the following herbs at a ratio of 9:6:6:6:6:6:6:6:6:6:6:6:6:6:6 (**Table 1**): *Angelica pubescens* Maxim. (Duhuo in Chinese) 9 g, *Asarum heterotropoides* F.Schmidt (Xixin in Chinese) 6 g, *Saposhnikovia divaricata* (Turcz. ex Ledeb.) Schischk. (Fangfeng in Chinese) 6 g, *Neolitsea cassia* (L.) Kosterm (Rougui in Chinese) 6 g, *Gentiana macrophylla* Pall. (Qinjiao in Chinese) 6 g, *Taxillus chinensis* (DC.) Danser (Sangjisheng in Chinese) 6 g, *Eucommia ulmoides* Oliv. (Duzhong in Chinese) 6 g, Cyathula officinalis K.C.Kuan, (Chuanniuxi in Chinese) 6 g, *Paeonia lactiflora* Pall. (Baishao in Chinese) 6 g, *Rehmannia glutinosa* (Gaertn.) DC. (Dihuang in Chinese) 6 g, *Angelica sinensis* (Oliv.) Diels (Danggui in Chinese) 6 g, *Panax ginseng* C.A.Mey. (Renshen in Chinses) 6 g, *Conioselinum anthriscoides ’Chuanxiong’* (Chuanxiong in Chinese) 6 g, *Smilax glabra* Roxb. (Fuling in Chinese) 6 g, and *Glycyrrhiza glabra* L. (Gancao in Chinese) 6 g. The names of all constituent herbs were verified from http://mpns.kew.org on June 12th, 2023. All constituent herbs were obtained from the pharmacy department of the Hospital of Chengdu University of Traditional Chinese Medicine.

**Table 1:** Composition of DHJSD.

Chinese name	Latin name	Weight (g)	Medicinal part
Duhuo	Radix Angelicae Biseratae	9	Taproot
Xixin	Asari Radix Et Rhizoma	6	Root
Fangfeng	Saposhnikoviae Radix	6	Taproot
Rougui	Cinnanmomi Cortex	6	Bark
Qinjiao	Gentiana Macrophylla Pall	6	Root
Sangjisheng	Herba Taxilli	6	Stem
Duzhong	Eucommiae Cortex	6	Bark
Chuanniuxi	Cyathulae Radix	6	Taproot
Baishao	Paeoniae Radix Alba	6	Taproot
Dihuang	Rehmanniae Radix Praeparata	6	Rhizome
Danggui	Angelicae Sinensis Radix	6	Taproot
Renshen	Panax Ginseng C. A. Mey	6	Root
Chuanxiong	Chuanxiong Rhizoma	6	Root
Fuling	Poria Cocos(Schw.) Wolf.	6	Fungal mass
Gancao	Licorice	6	Root

DHJSD at doses of 7*93g, 13*93g, and 20*93g was soaked in 2.5L, 3.5L and 5.5L of water for 60 minutes respectively, and then boiled. Then, the solutions were concentrated to 1085mL, 1007.5mL, and 980mL at 50°C to obtain low-dose (0.6g/mL), medium-dose (1.2g/mL), and high-dose (1.9g/mL) of DHJSD, respectively. The DHJSD solutions obtained were stored in sterile bottles at 4°C for future use.

### Animal experiments

#### Establishment of OA rat model

The animal study was approved by the Experimental Animal Ethics Committee of Hospital of Chengdu University of Traditional Chinese Medicine (No.2023DL-005). A total of 25 adult, male Wistar rats (weight:198g-220 g) were obtained from Dossy (Chengdu, Sichuan, China). The rats were acclimatized for 1 week at 24±2°C under a 12-h light/dark cycle and were provided free access to food and water. Subsequently, rats were randomized into the sham control group (where only the joint cavity was exposed; n = 5), model control group (n = 5), high-dose (HD) group (n = 5), medium-dose (MD) group (n = 5), low-dose (LD) group (n = 5).

**Fig 1:**
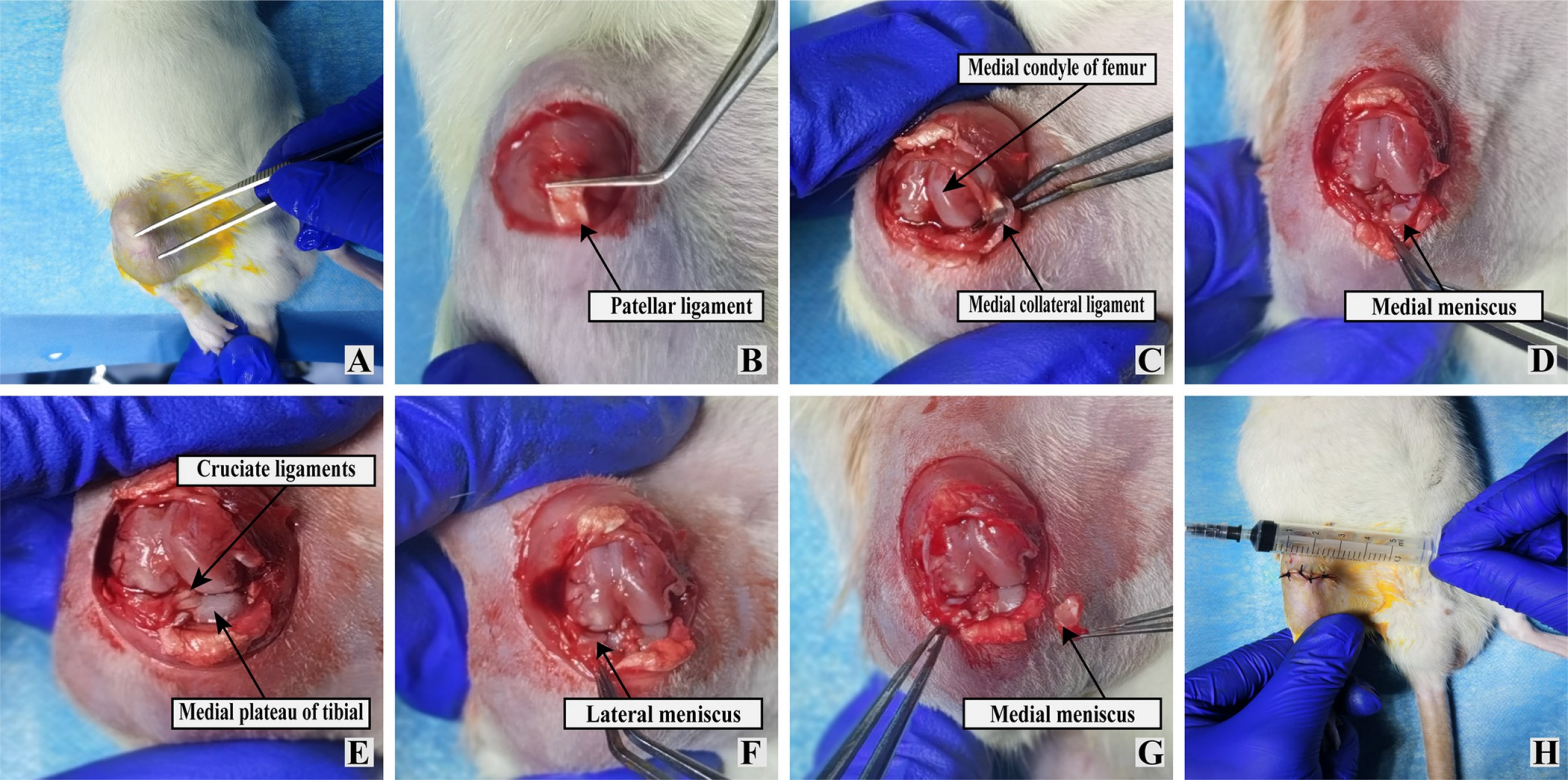
Establishment of rat KOA model using the Hulth method. (A) Incision site; (B) exposure of the patellar ligament; (C) cutting of the patellar ligament and exposure of the medial collateral ligament; (D) cutting of the medial collateral ligament and exposure of the medial meniscus; (E) removal of the medial meniscus and exposure of the cruciate ligament; (F) cutting of the cruciate ligament and retention of the lateral meniscus; (G) image showing cut medial collateral ligament and cruciate ligaments, removed medial meniscus, and fully retained lateral meniscus; (H) suturing of incision.

The rat OA model was established using the Hulth method after inducing anesthesia using intraperitoneal pentobarbital sodium [30]. As showed in **Fig 1**, OA was induced by incising the medial side of the right posterior knee joint of anesthetized rats. The muscles and ligaments were then separated to expose the joint cavity. The medial collateral ligament, anterior and posterior cruciate ligament, and the medial meniscus were cut off. Subsequently, the incision was sutured, and each rat was intramuscularly administered penicillin (400 000 U/d) for 3 consecutive days and housed in the cage.

#### Animal treatments

The HD, MD, and LD groups were administered with 1.5 mL/100 g∙day of high, medium, and low-dose DHJSD, respectively, via gavage twice daily for 4 weeks from the first day after operation. The sham and model control groups received saline at the same dosage as DHJSD. After treatment, all rats were euthanized in accordance with the ethical guidelines for animal welfare, and blood and knee joint specimens were obtained for further assessments.

### Histological analysis and macroscopic observation

Cartilage morphology was evaluated using hematoxylin-eosin, Masson, immunohistochemical, and TRAP staining via light microscopy. The autophagosomes in chondrocytes were observed using transmission electronic microscopy.

### Enzyme-linked immunosorbent assay

The levels of MMP-13 and IL-1β in rat serum were determined using the enzyme-linked immunosorbent assay kit (IL-1β: ZC-36391, Spec. 48 Test; MMP-13: ZC-36747, Spec. 48 Test, Zhuocai Biotechnology, Shanghai, China). Quantification was performed by measuring the absorbance at 450 nm using a microplate reader (SpectraMAX Plus384, Molecular Devices, USA).

### Preparation of DHJSD-serum

Twenty adult, male Wistar rats (weight: 198g-220 g) were obtained from Chengdu Dossy Experimental Animals Co. LTD (Sichuan, China). After 1 week of acclimatizing under the aforementioned conditions, the animals were randomized into two groups: group A (n = 10) received 1.5 mL/100 g∙day of DHJSD twice daily for 1 week and group B (n = 10) received the same dosage of normal saline. One hour after the last administration, blood was collected from the abdominal aorta, and the serum was separated by centrifugation. The obtained serum was inactivated in a water bath at 56°C for 30 minutes, packaged, and frozen at -80°C until further use. Subsequently, all rats were euthanized in accordance with the ethical guidelines for animal welfare.

### Quality control of DHJSD

DHJSD components were detected using an LC-MS/MS system, ultra-high performance liquid chromatograph (Nexera UHPLC LC-30A, SHIMADZU, Japan) with mass spectrometer (TripleTOF5600+, AB SCIEX™). The liquid chromatography conditions were as follows: ChromCore 120 C18 Column (1.8μm 2.1mm*150mm), column temperature of 40°C, mobile phase A of 0.1% formic acid and mobile phase B of 100% ACN, flow rate of 0.3mL/min, and analysis time of 21min. Detection was performed using electrospray ionization (ESI) in negative ion mode. The ESI source conditions were as follows: Ion Source Gas1 (Gas 1): 50, Ion Source Gas2 (Gas 2): 50, Curtain Gas (CUR): 25, temperature of 450°C (negative ion), voltage of 4400V (negative ion), TOF MS scan range of 100-1200Da, product ion scan range of 50-1000Da, TOF MS scan accumulation time of 0.2s, product ion scan accumulation time of 0.01s. The second-level mass spectrometry was obtained using information-dependent acquisition (IDA) in high sensitivity mode, with a declustering potential (DP) of ±60V and a collision energy of 35±15eV.

### PTEN siRNA transfection

The chondrocytes were transfected with a specific PTEN siRNA obtained from RiboBio (Guangzhou, China). For this, 15 μL of siRNA was diluted with 360 μL 1X riboFECT^TM^CP Buffer in a 1.5 mL centrifuge tube and gently mixed. To this, 36 μL of the riboFECT^TM^CP Reagent was added, gently blown, and mixed, followed by incubation at room temperature for 15 min to prepare the riboFECT^TM^CP transfection complex (siRNA concentration: 50 nmol). Chondrocytes were seeded in a six-well plate. After ensuring cell adhesion to the wall, the supernatant in the wells was removed by suction. Subsequently, the riboFECT^TM^CP transfection complex was added to an appropriate amount of the complete medium without double antibody, mixed, added to the well plate, and then incubated at 37°C and 5% CO_2_ for further use.

### Cell treatment and CCK-8 assay

The CCK-8 assay was used to determine the optimal concentration of DHJSD-serum (Ds) obtained using four doses (0%, 5%, 10%, 15%, and 20%). The CCK-8 kit (B3350A, 21169949, Spec. 5*100T, Biosharp, Anhui, China) was used to analyze the cell proliferation rate, in accordance with the manufacturer’s instructions. The primary rat chondrocytes (Rat-iCell-s003, iCell) (5×104/mL, 100 μL/ well) in each group (4 wells in each group) were seeded in 96-well plates (edge wells were filled with sterile phosphate-buffered saline) and cultured for 48 h at 37°C with 5% CO_2_. After 48 h of treatment, the supernatant was aspirated and discarded. Subsequently, the CCK-8 reagent was diluted with a serum-free medium at a ratio of 1:10, and 110 μL of the diluted CCK-8 solution/well was added to the plates. The culture plate was then gently shaken several times and incubated further for 2 h at 37°C and 5% CO_2_. The absorbance values of each well were measured at 450 nm using a microplate reader.

### Measurement of cell apoptosis

The cells in each group were collected and resuspended in 500 μL binding Buffer. The 5 μL of Annexin V-APC/PI (Kit: KGA1030, KeyGEN, Jiangsu, China) was added to blow gently, followed by the addition of 5 μL of propidium iodide. The reaction was conducted at room temperature in the dark for 15min, following which apoptosis was detected and analyzed.

### Determination of intracellular zinc levels

The sample was placed in a Teflon vessel according to the grouping and concentrated nitric acid (5 mL) was added. Subsequently, the sample was digested using a microwave digestion apparatus (Shanghai, China), as per the standard protocol of the machine. Using the tuning fluid, instrument parameters were adjusted and optimized before each experiment to meet the requirements of sensitivity, resolution, and stability. The analysis was conducted in the CCT (He/O_2_) mode. The main working parameters and acquisition conditions of the instrument are listed below [31,32]. Tuning mode: STD/KED; dwell time: 0.1 s; peristaltic pump speed: 40 rpm; sample introduction time: 40 s; plasma power: 1550 W; sampling depth: 5.0 mm; nebulizer flow:0.98 L/min; cool flow:14.0 L/min; auxilliary flow:0.8 L/min; spray chamber temperature: 2.7°C; torch horizontal position: 0.16 mm; torch vertical position: -0.53 mm; helium flow: 4.55 mL/min; oxygen flow: 0.3125 mL/min; D1 lens: -350 V; and D2 lens: -350 V; repeat times: 3 times. The reference, multi-element calibration standard, and internal standard were purchased from the National Center of Analysis and Testing for Nonferrous Metals and Electronic Materials (Beijing, China).

### Real-time polymerase chain reaction

Total cellular RNA was isolated using animal total RNA isolation kit (RE-03014, Spec. 200, Foregene, China) according to the manufacture’s instruction. Complementary DNA (cDNA) was reverse-transcribed with PrimeScript-RT reagent kit (RR047A, Spec.100, Takara, Japan).The mRNA levels of Beclin-1, Bax, Bcl-2 and PTEN were detected by RT-PCR with TB Green TM Premix Ex TaqTM Ⅱ(Tli RNaseH Plus) (RR820A, Spec. 200, Takara, Japan). The complete gene sequences showed in **Table 2** were searched from the National Center for Biotechnology Information (NCBI) database, and the specific primers were designed and screened by Primer Premier software. All primers were designed and synthesized by Sangon Bioengineering Technology(Shanghai, China), and purified by ULTRAPAGE. Data were analyzed by 2^−ΔΔCT^ method.

**Table 2:** Primer sequence.

Gene	Forward	Reverse
**β-actin**	gggaaatcgtgcgtgacatt	gcggcagtggccatctc
**Beclin-1**	aaggagttgccgttgtactgttctg	tgcctccagtgtcttcaatcttgc
**LC3II/I**	gagcgagttggtcaagatcatccg	gatgtcagcgatgggtgtggatac
**Bax**	agacacctgagctgaccttggag	ttcatcgccaattcgcctgagac
**Bcl-2**	tggagagcgtcaacagggagatg	acagccaggagaaatcaaacagagg
**PTEN**	tttgaagaccataacccaccacagc	cattacaccagtccgtcctttccc

### Western blot analysis

The protein levels of PTEN, Akt, p-Akt, mTOR, p-mTOR, Bax, Bcl-2, Beclin-1 and LC3-II/I in chondrocytes were detected using western blotting. Total cellular proteins were extracted using RIPA lysates. The proteins were transferred to PVDF membrane by wet rotation method and blocked at room temperature for 1 h. The primary antibody was incubated overnight and the membrane was washed 3 times with TBST for 5 min each time. The secondary antibody was incubated at room temperature for 2 h, and the membrane was washed 3 times with TBST for 10 min each time. Then the ECL developer solution was uniformly added to the membrane for exposure development. The bands were scanned by exposure using Tianneng GIS chassis control software V2.0, and the results were expressed as the relative expression of the target protein.

### Statistical analyses

SPSS 26 (IBM® SPSS® Statistics) was used to analyze the data in this study, and figures were plotted using GraphPad Prism v9.5.1 (GraphPad Software, Inc.). All data are presented as the means ± standard error of means, and all experiments were performed in triplicate. Differences among groups were analyzed by one-way analysis of variance, followed by Fisher’s post hoc test. Differences between two groups were analyzed using the unpaired, two-tailed Student’s *t*-test. Results with P<0.05 were considered statistically significant.

## Results

### Qualitative analysis of DHJSD components

Liquid-chromatography tandem mass spectrometric analysis of DHJSD revealed 173 compounds. Compounds with a total score of 100 as well as their formula, ontology, reference and retention time are listed as in **Table 3**. Positive and negative ion chromatograms were provided in the Supplementary Material (S1 Fig).

**Table 3:** Compounds in DHJSD with a total score of 100.

Formula	Ontology	Reference (m/z)	RT (min)	Total score
C_17_H_22_O_11_	Iridoid glycosides	401.10849	6.1149	100
C_21_H_44_NO_6_p	Glycerophosphoethanolamines	436.28336	15.86822	100
C_20_H_36_N_4_O_8_	N-acyl amines	461.26059	17.3248	100
C_9_H_17_NO_5_	Fatty acids	218.1032	1.7611	100
C_6_H_12_O_6_	Monosaccharides	179.056	1.239233	100
C_5_H_14_NO	Cholines	104.10699	19.25602	100
C_8_H_11_NO_3_	Pyridoxines	170.05896	5.367417	100
C_15_H_22_O_3_	Tertiary alcohols	249.08504	6.836583	100
C_33_H_40_N_2_O_9_	Yohimbine alkaloids	609.33337	16.61178	100
C_5_H_4_N_4_	Purines and purine derivatives	119.036	1.36025	100
C_9_H_10_O_3_	Alkyl-phenylketones	165.05571	7.313766	100

### Effects of DHJSD on MMP-13 and IL-1β in KOA rats

Studies have shown that DHJSD inhibits the production of inflammatory factors, including interleukins and matrix metalloproteinases (MMPs) [33,34]. However, there is a lack of detailed research on the mechanism via which DHJSD in protects articular cartilage in KOA.

**Fig 2:**
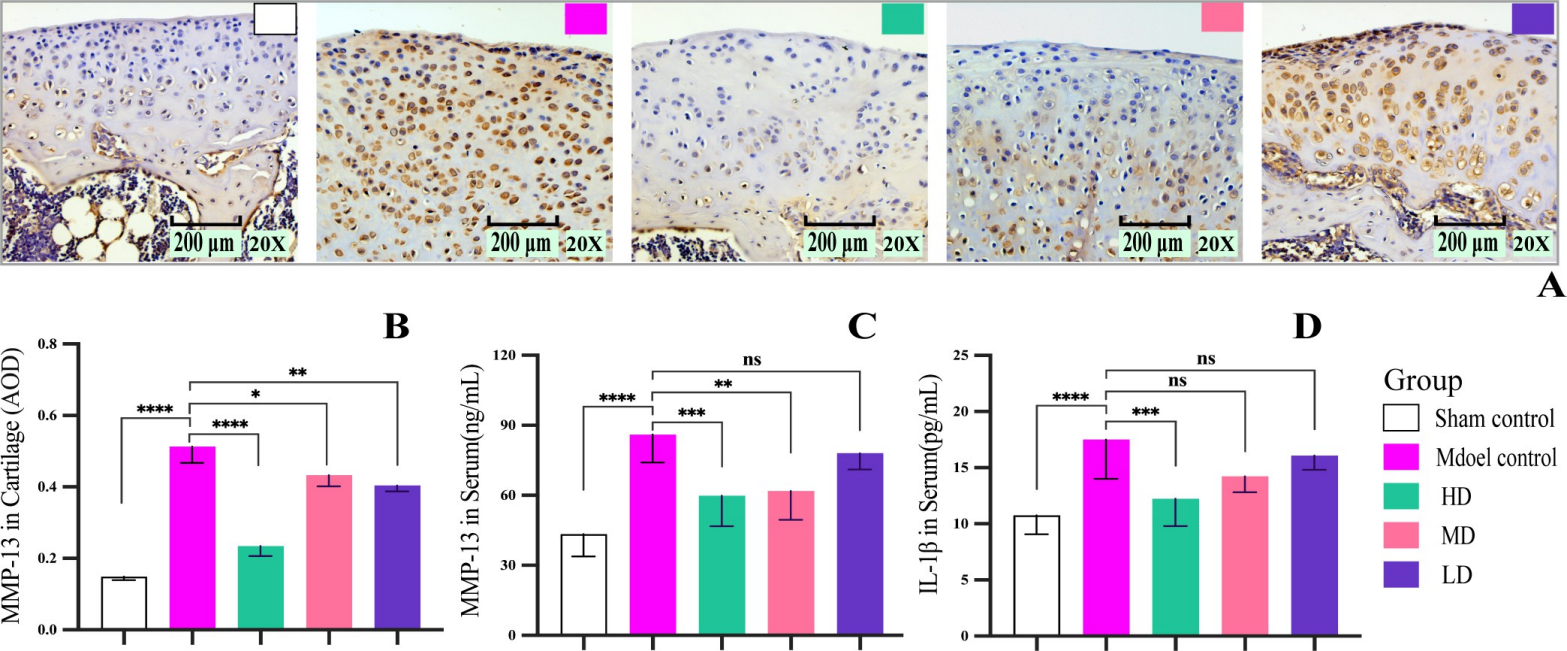
DHJSD decreased MMP-13 and IL-1β levels in KOA rats. (A) Representative images of immunohistochemical staining of MMP-13 in each group. (B) Quantitative analysis of MMP-13 in the cartilage matrix. (C, D) Quantitative analysis of MMP-13 and IL-1β in serum. Data are presented as the means ± standard deviations (n = 3). *p < 0.05, **p < 0.01, ***p < 0.001, ****p < 0.0001,^ns^p > 0.05.

In the present study, the rat KOA model was established using the classic Hulth method. Unlike previous studies, the current study administered DHJSD to rats 24 h after surgery. Regarding the inflammatory factors (Fig 2), the levels of MMP-13 and IL-1β in the serum, as well as the expression area of MMP-13 in the cartilage matrix were increased in four weeks after KOA induction. However, pretreatment with DHJSD decreased these levels by varying degrees in KOA rats, and the differences were statistically significant. These results further confirm that DHJSD can reduce the expression of MMP-13 in the cartilage matrix and serum of rats with KOA.

### DHJSD alleviated cartilage pathological characteristics in KOA rats

**Fig 3:**
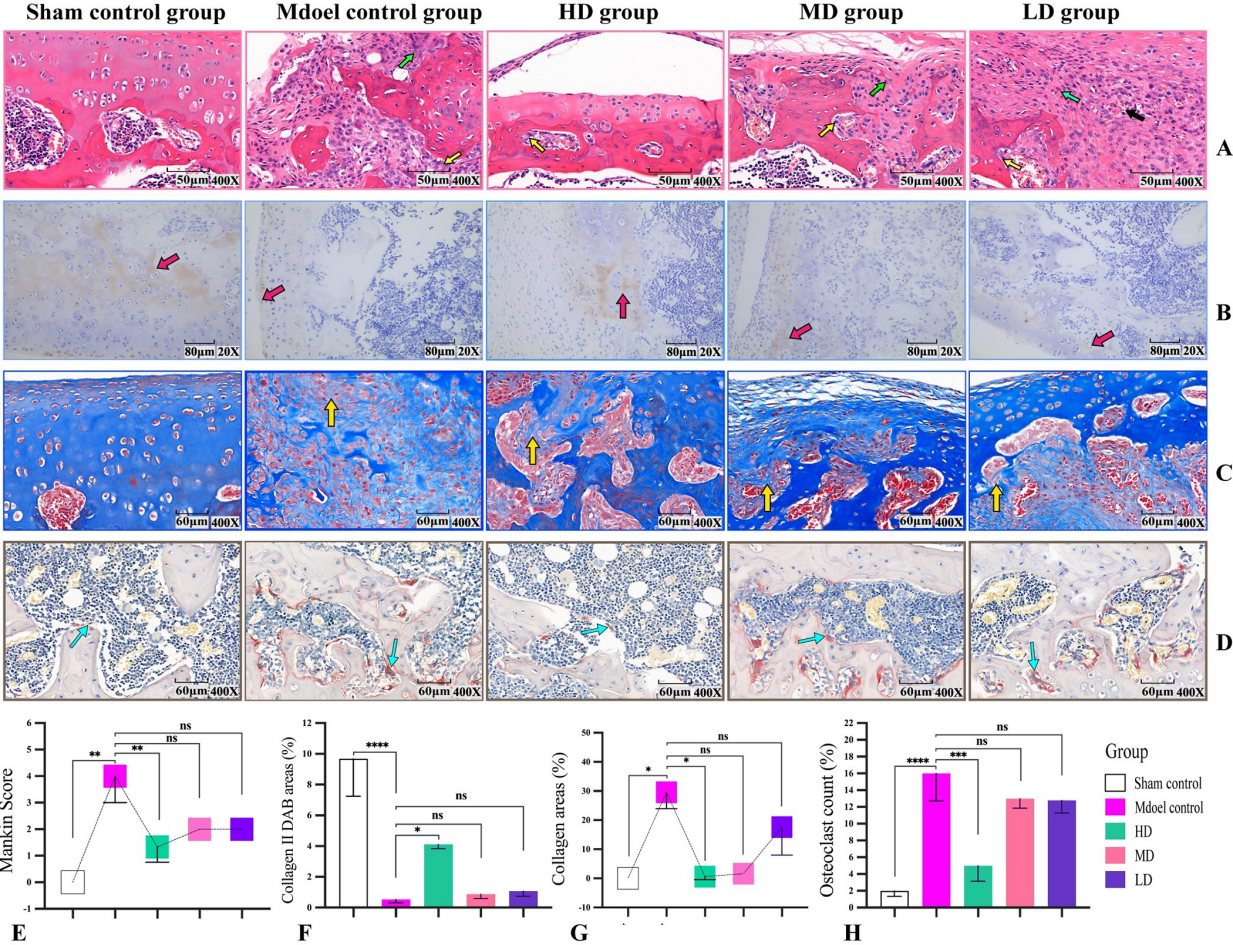
DHJSD alleviated the pathological characteristics of the cartilage in KOA rats. (A) Representative hematoxylin-eosin images of the rat knee joints in each group. (B) Representative images of immunohistochemical staining of collagen II in each group. (C) Representative images of Masson staining of collagenous fiber in each group. (D) Representative images of TRAP staining of osteoclasts in each group. (E–H) Quantitative analysis of hematoxylin-eosin, immunohistochemical, Masson, and TRAP staining. Data are presented as the means ± standard deviations (n = 3). *p < 0.05, **p < 0.01, ***p < 0.001, ****p < 0.0001,^ns^p > 0.05.

Next, we examined the effects of DHJSD on cartilage morphology in rats. Hematoxylin-eosin staining, Masson staining, immunohistochemical staining and TRAP staining were used to evaluate the pathological characteristics of cartilage. After 4 weeks of KOA induction, the model group exhibited severe loss of type II collagen, increased cartilage destruction, osteoclast formation, and collagen fiber formation, and higher Mankin score than the sham control group (**Fig 3**). However, pretreatment with HD DHJSD resulted in only mild changes. The results indicated that DHJSD had a certain inhibitory effect on cartilage destruction.

### DHJSD increased autophagy and inhibited apoptosis in KOA rats

**Fig 4:**
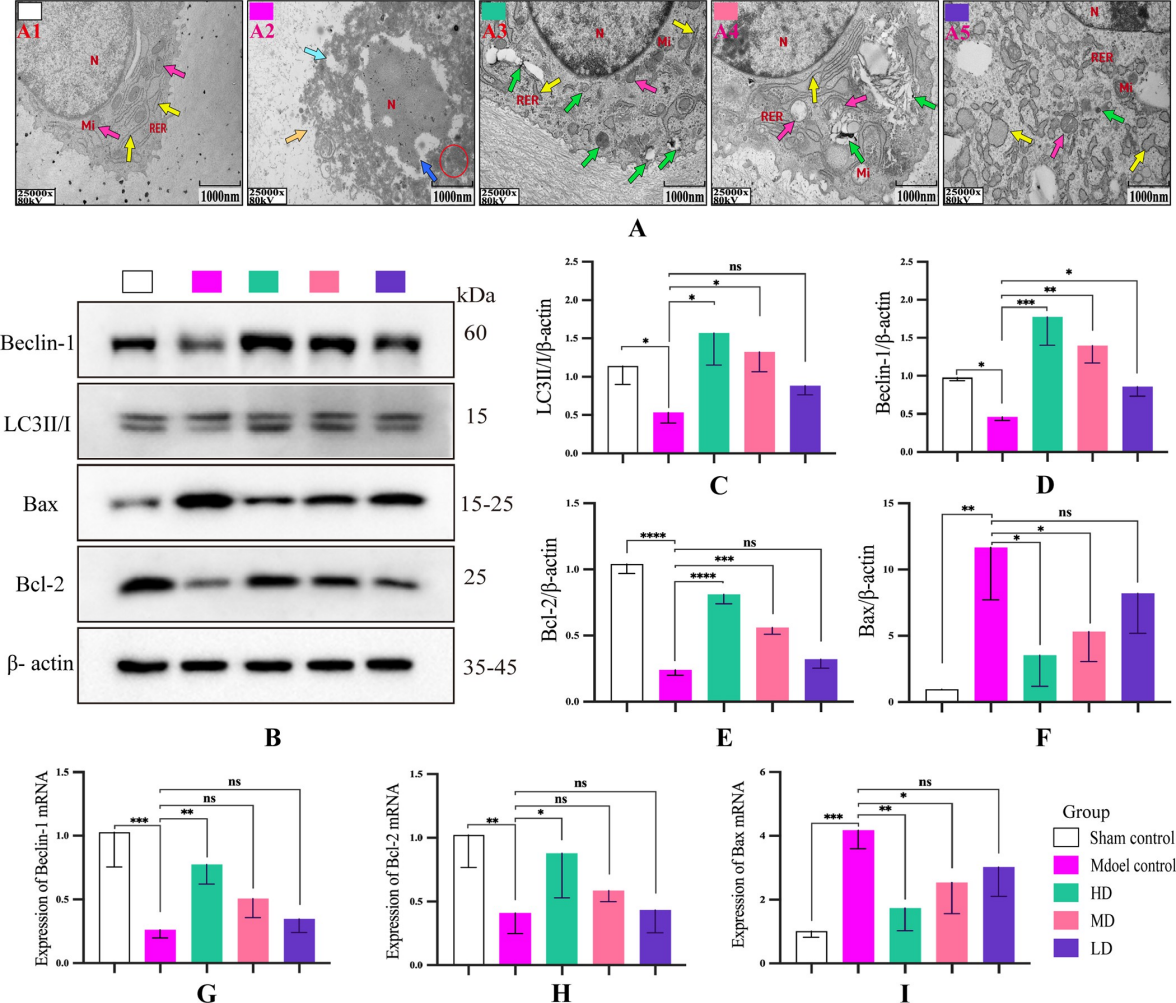
Effect of DHJSD on autophagy and apoptosis in KOA rats. (A) Representative transmission electron microscopy images of rat chondrocytes in each group. (B) Bands of autophagy- and apoptosis- related indicators, including LC3II/I, Beclin-1, Bax, and Bcl-2. (C–F) Quantitative analysis of LC3II/I, Beclin-1, Bax, and Bcl-2. (G–I) Quantitative analysis of mRNA expression of Beclin-1, Bax, and Bcl-2. Data are presented as the means ± standard deviations (n = 3). *p < 0.05, **p < 0.01, ***p < 0.001, ****p < 0.0001,^ns^p > 0.05.

As shown in **Fig 4A,** the model group demonstrated a discontinuous cell membrane, concentrated cytoplasm, and lysed nucleus; moreover, the cytoplasmic content was missing in some areas, and the autophagosomes were hardly observed. Although the HD group demonstrated dilated rough endoplasmic reticulum in some cells, the morphological structure of cells and mitochondria was almost normal, with more autophagosomes in the cytoplasm. Treatment with DHJSD demonstrated a significant, dose-dependent increase in autophagic vesicles in the cytoplasm. At the same time, DHJSD significantly increased the protein levels of LC3 and Beclin-1 and the mRNA expression of Beclin-1 (**Fig 4C, 4D and 4G**). In contrast, DHJSD decreased the protein level and mRNA expression of Bax and Bcl-2 (**Fig 4E–4I**). Taken together, DHJSD can increase the level of autophagy and inhibit the apoptosis of articular chondrocytes in KOA rats.

### DHJSD modulated the PTEN/Akt/mTOR signaling pathway in KOA rats

**Fig 5:**
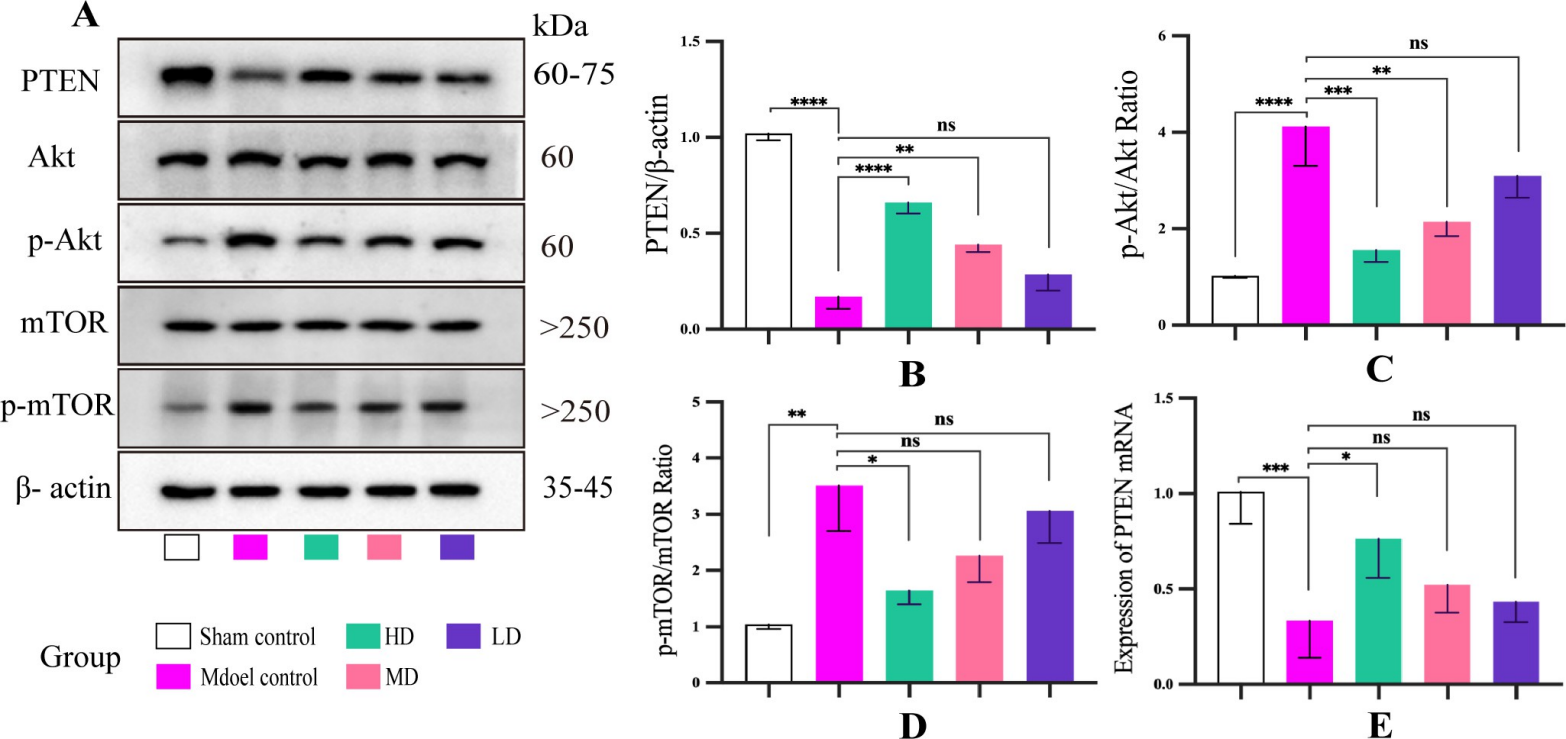
Effect of DHJSD on PTEN/Akt/mTOR signaling pathway in KOA rats. (A) Western blotting analysis of PTEN, Akt, p-Akt, mTOR, and p-mTOR. (B-D) Quantitative analysis of western blotting. (E) Quantitative analysis of mRNA expression of PTEN. Data are presented as the means ± standard deviations (n = 3). *p < 0.05, **p < 0.01, ***p < 0.001, ****p < 0.0001, ^ns^p > 0.05.

It is well known that the Akt/mTOR signaling axis is closely related to autophagy and that PTEN has a direct regulatory effect on this signaling pathway. We hypothesized that DHJSD-mediated increase in chondrocyte autophagy may be mediated via the PTEN/Akt/mTOR pathway. To verify this hypothesis, the protein levels of PTEN, Akt, p-Akt, mTOR, and p-mTOR and the mRNA expression of PTEN were determined. Western blotting and RT-PCR findings indicated that after 4 weeks of DHJSD treatment in the HD group, *PTEN* expression was markedly increased, but p-Akt and p-mTOR expression was significantly decreased (**Fig 5B–5E**). These findings indicate that DHJSD promotes chondrocyte autophagy by regulating the PTEN/Akt/mTOR pathway.

### DHJSD increased autophagy and reduced apoptosis and zinc levels *in vitro*

**Fig 6:**
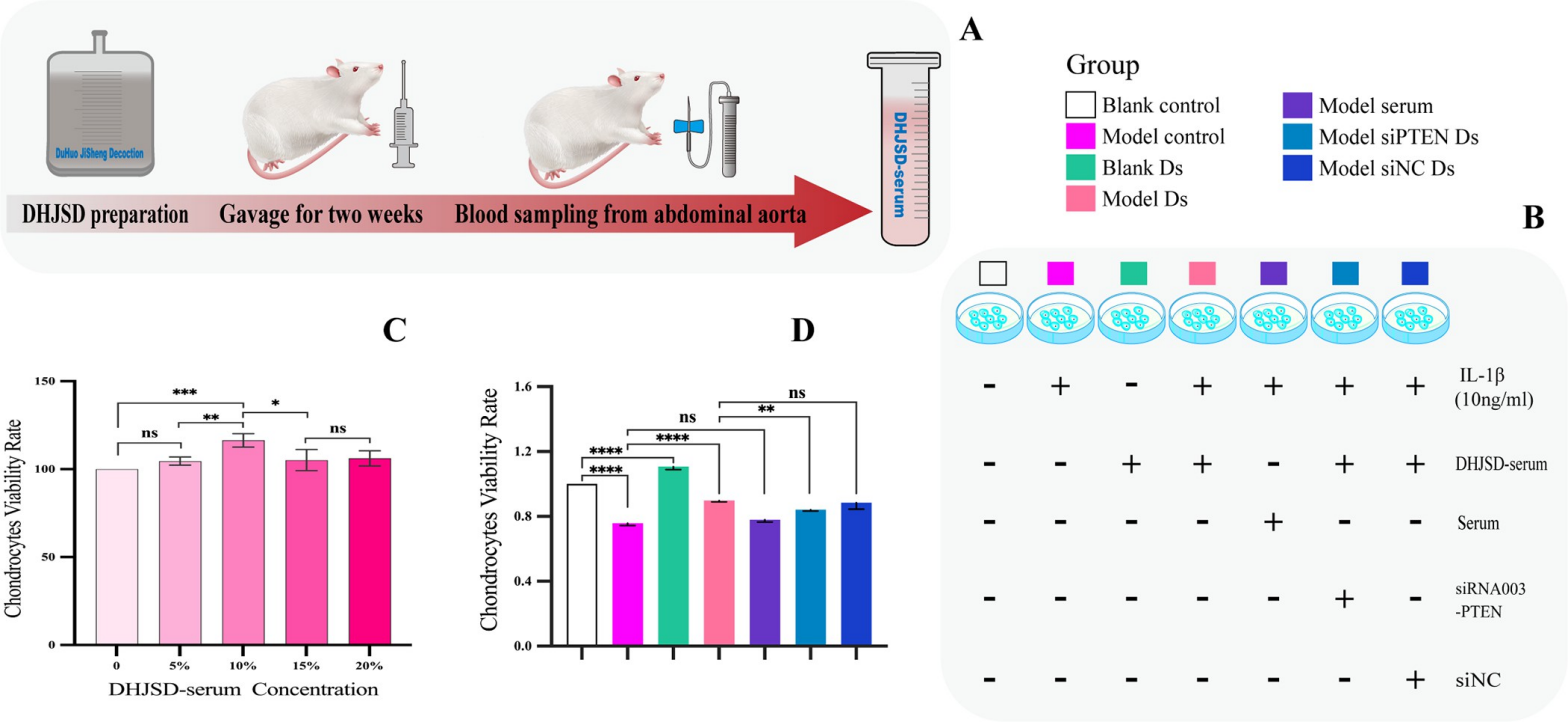
Screening of the optimal Ds concentration and the effect of Ds on the chondrocyte viability. (A) Schematic representation of Ds preparation. (B) Schematic representation of cell grouping and pretreatment. (C, D) Quantitative analysis of chondrocyte viability rate.

The results of *in vivo* experiments confirmed that DHJSD improved cartilage degeneration in KOA rats by inducing autophagy in chondrocytes. Accordingly, we further attempted to clarify the effects of DHJSD on the chondrocytes using *in vitro* experiments. Based on *in vivo* data, HD DHJSD was used to prepare the Ds, as shown in **Fig 6A**.

To first determine the optimal Ds concentration for the treatment of cells *in vitro*, chondrocytes were cultured with Ds gradient for 48 h, followed by cell viability analysis using the CCK-8 assay. The results showed that the cell viability of the group treated with 10% Ds for 48 hours was greater than that of the other four groups (**Fig 6C**). Chondrocyte viability rate was inhibited by 10 ng/mL of IL-1β, and this effect was reversed by pretreatment with 10% Ds. However, this effect was significantly attenuated after *PTEN* silencing. Therefore, 10% Ds was used for subsequent experimental studies (**Fig 6D**).

**Fig 7:**
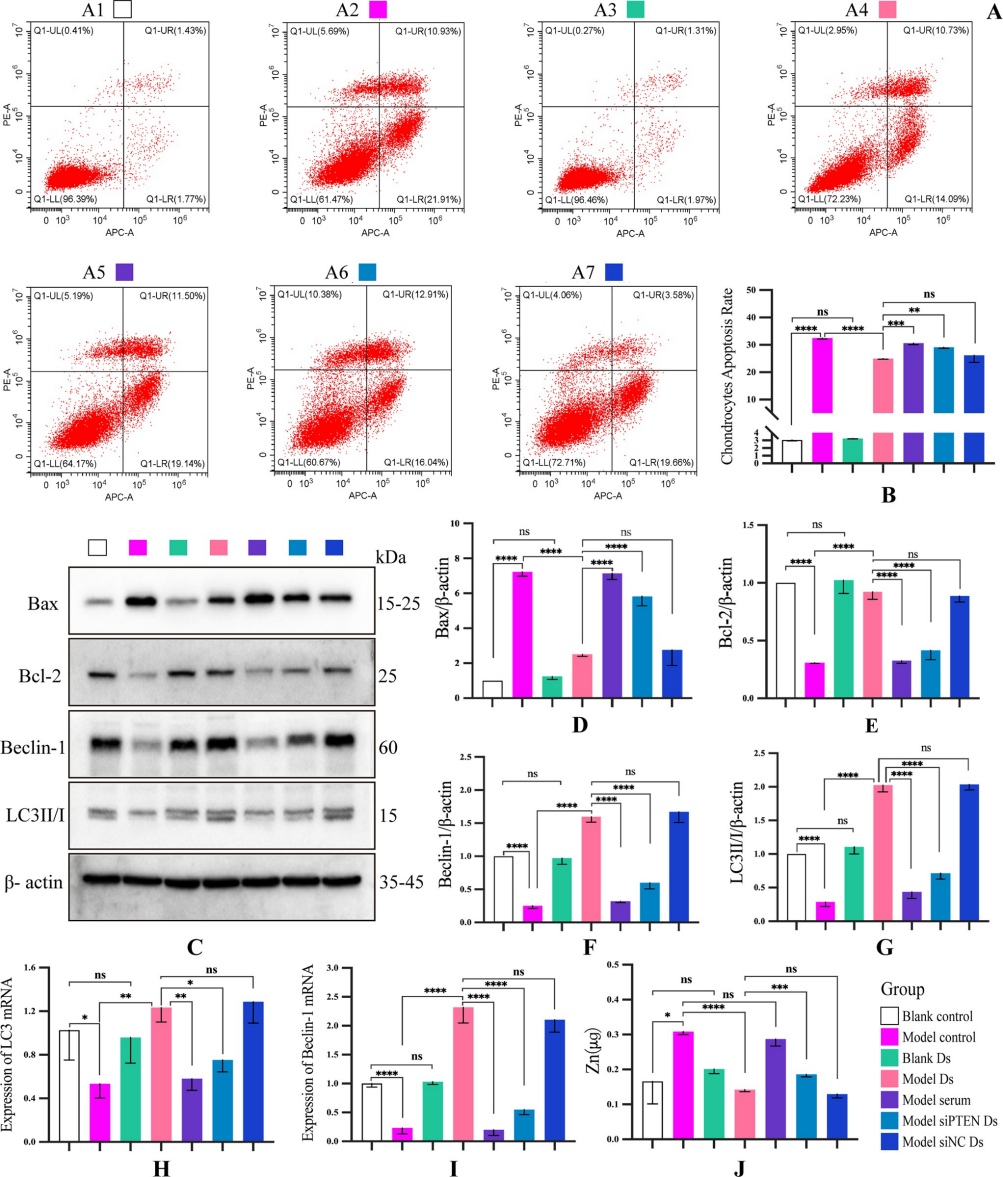
Effect of DHJSD on *in vitro* autophagy, apoptosis and zinc levels in chondrocytes. (A) Representative images of scatter plots of chondrocyte apoptosis determined using flow cytometry. (B) Quantitative analysis of chondrocytes apoptosis. (C) Western blotting analysis of Bax, Bcl-2, Beclin-1,and LC3II/I. (D-G) Quantitative analysis of western blotting findings. (H, I) Quantitative analysis of mRNA expression of LC3, and Beclin-1. (J) Quantitative analysis of zinc levels in chondrocytes. Data are presented as the means ± standard deviations (n = 3). *p < 0.05, **p < 0.01, ***p < 0.001, ****p < 0.0001, ^ns^p >0.05.

Next, we examined cell apoptosis in each group using flow cytometry (**Fig 7A**). The level of apoptosis was reduced significantly after 48-h treatment with Ds, whereas this effect was significantly depressed after *PTEN* silencing (**Fig 7B)**. Western blotting and RT-PCR analysis of apoptosis and autophagy markers **(Fig 7D and 7E)** revealed that Bax and Bcl-2 protein levels were significantly upregulated after 48-h IL-1β treatment, and this effect was reversed by 48-h pretreatment with DHJSD-serum. Furthermore, treatment with specific siRNA PTEN silenced *PTEN* expression and inhibited the anti-apoptotic effect of Ds. The protein levels of LC3 and Beclin-1 were significantly decreased after IL-1β treatment, and the effect was reversed by pretreatment with Ds. Additionally, *PTEN* silencing weakened Ds-induced increase in the level of autophagy in chondrocytes (**Fig 7F–7I**).

Reportedly, DHJSD can prevent the degradation of the cartilage matrix by inhibiting MMPs, as revealed in *in vivo* experiments. Furthermore, zinc is an essential cofactor for a variety of matrix metalloproteinases and is involved in the metabolic homeostasis of cartilage matrix [35,36]. Moreover, previous studies have found that DHJSD can decrease zinc levels in chondrocytes and inhibit the expression of MMP-13 and other proteins [19,33]. In addition, autophagy has been found to be closely related to intracellular zinc homeostasis [16–18]. Therefore, we hypothesized that promoting the autophagy of chondrocytes by DHJSD was also related to the intracellular zinc levels. To verify the hypothesis, ICP-MS/MS was used to measure zinc levels in chondrocytes. The results showed that IL-1β treatment increased zinc levels in chondrocytes. However, treatment with Ds significantly reduced intracellular zinc levels, and this effect was significantly reversed after *PTEN* silencing (**Fig 7J**). Collectively, these findings indicate that DHJSD decreases zinc levels by upregulating autophagy.

### DHJSD regulated the PTEN/Akt/mTOR signaling pathway *in vitro*

Animal experiments have confirmed that DHJSD can enhance chondrocyte autophagy via PTEN/Akt/mTOR signaling pathway. Hence, we attempted to further elucidate this mechanism *in vitro*.

**Fig 8:**
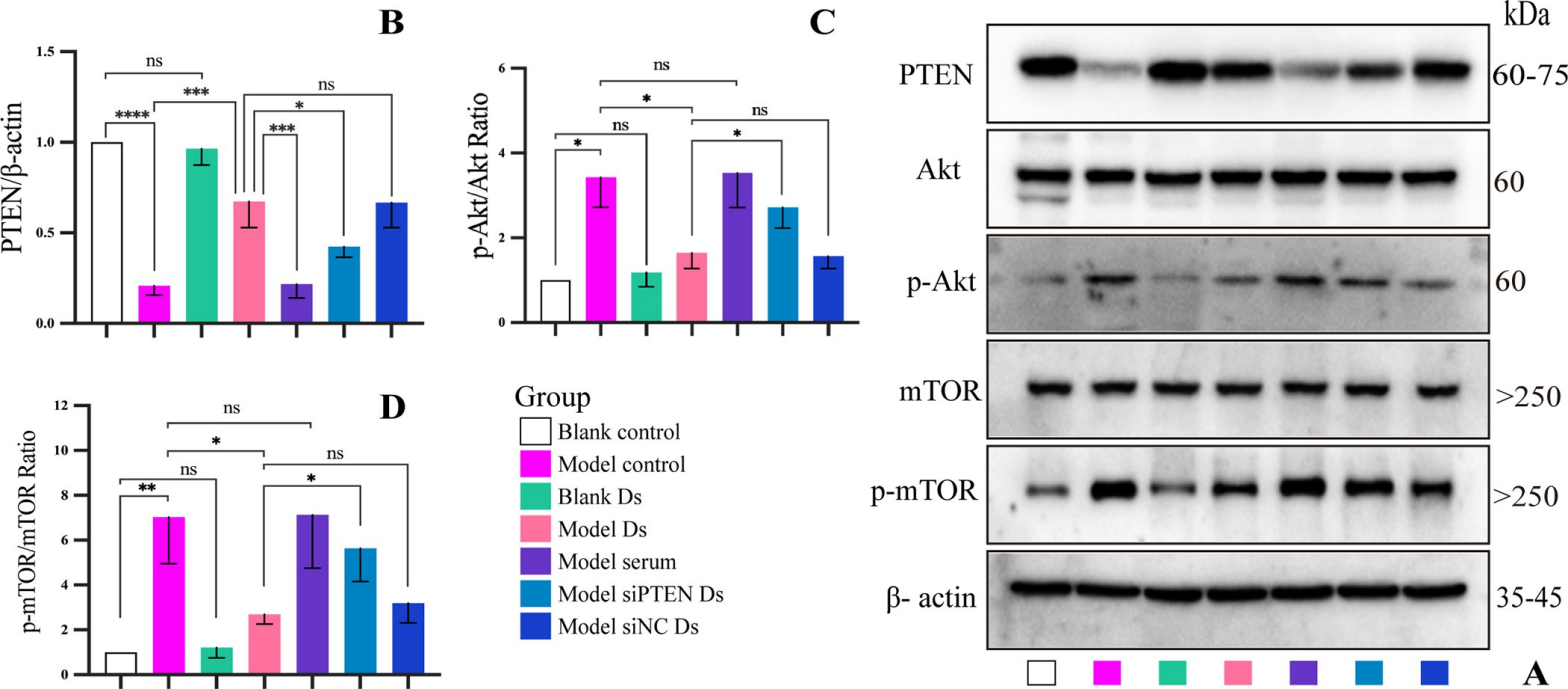
Effect of DHJSD on PTEN/Akt/mTOR signaling pathway in chondrocytes *in vitro*. (A) Western blotting analysis of PTEN, Akt, p-Akt, mTOR, and p-mTOR. (B-D) Quantitative analysis of western blotting data. Data are presented as the means ± standard deviations (n = 3). *p < 0.05, **p < 0.01, ***p < 0.001, ****p < 0.0001, ^ns^p > 0.05.

Identification of the protein levels of PTEN, p-Akt, Akt, p-mTOR, and mTOR using western blotting revealed that pretreatment with Ds promoted *PTEN* expression and reduced Akt and mTOR phosphorylation in IL-1β-stimulated chondrocytes. However, silencing *PTEN* reduced the inhibitory effect of Ds on Akt and mTOR phosphorylation. Notably, Ds demonstrated no significant effect on *PTEN* expression and Akt and mTOR phosphorylation levels in normal chondrocytes. These findings were additionally confirmed in *in vivo* experiments (**Fig 8)**.

## Discussion

The four major pathological and etiological characteristics of KOA include inflammatory cytokine cascade activation, chondrocyte apoptosis, imbalanced subchondral bone remodeling, and extracellular matrix degradation [37]. The majority of the research for studying cartilage degeneration is directed at subchondral bone remodeling, delaying chondrocyte apoptosis, and inhibiting matrix degradation. Currently, non-steroidal anti-inflammatory drugs are the most widely used and effective drugs in the conventional treatment of KOA [38,39]. However, the long-term use of these drugs is associated with side effects such as gastrointestinal ulcers and cardiovascular adverse events [40], which are concerning. Moreover, the application of specific treatments for OA, such as glucosamine, hyaluronic acid, and platelet-rich plasma injections, are controversial and either conflict with the guidelines or are not recommended owing to a lack of evidence-based findings regarding the long-term efficacy [41]. Therefore, in the current situation, the use of natural compounds, including traditional Chinese medicine formulas, is one of the important directions for the prevention and treatment of KOA. In this regard, DHJSD has a long history of use for the treatment of KOA; however, the mechanism underlying its action has not been fully elucidated.

The soft tissue mechanical abnormality is one of the factors initiating OA [42]. Therefore, the classic Hulth method was used to establish the KOA rat model in this study. Reportedly, IL-1β plays a key role in OA pathogenesis [43], and it has been well-established that IL-1β can be used to establish an *in vitro* cellular OA model. In the present study, both the Hulth method-induced KOA model (*in vivo*) and IL-1β-induced cellular KOA model (*in vitro*) substantially replicated human OA-like features. Moreover, treatment of KOA rats with DHJSD attenuated the OA-like features in rats.

The present study investigated the mechanism by which DHJSD prevents and treats KOA in an *in vivo* rat KOA model and *in vitro* IL-1β-induced chondrocytes. The results showed that DHJSD could inhibit IL-1β and MMP-13 levels, which was consistent with the results of previous studies [44], thus confirming the anti-inflammatory effect of DHJSD. In addition, the *in vivo* experiment revealed that DHJSD could alleviate the articular cartilage degradation, repress osteoclast formation, reduce collagen fiber deposition, and prevent type II collagen degradation in the articular cartilage. Moreover, DHJSD could upregulate the protein and mRNA levels of *PTEN*; inhibit the Akt/mTOR signaling pathway; and promote LC3 and Belcin-1 expression, inhibit Bax and Bcl-2 expression, and reduce zinc levels in chondrocytes.

Autophagy is a key important protective mechanism for cells to avoid self-apoptosis [45,46]. Reportedly, the PTEN/Akt signaling axis is closely related to autophagy [20,26,47]. In the present study, IL-1β treatment downregulated the expression of PTEN, LC3, and Beclin-1 and upregulated the phosphorylation of Akt and mTOR. In contrast, pretreatment of IL-1β-stimulated chondrocytes with Ds increased the expression of PTEN, LC3, and Beclin-1 and decreased the phosphorylation of Akt and mTOR. However, this effect was weakened following the silencing of *PTEN*. Furthermore, *in vivo* results demonstrated that the expression of PTEN, LC3, and Beclin-1 in the chondrocytes of KOA rats in the HD group were significantly higher than the expression in the model group, but the levels of p-Akt and p-mTOR were significantly lower than those in the model group. In addition, transmission electron microscopy findings revealed that the formation of autophagosomes in rat chondrocytes in the HD group was significantly higher than that in the model group. Therefore, it is reasonable to speculate that DHJSD can target *PTEN* to inhibit the Akt/mTOR signaling pathway and upregulate autophagy and LC3 and Beclin-1 protein levels in chondrocytes.

It is well established that MMP-13 plays an important role in the progression of OA [48–50], which is the pathological basis for the degradation of articular cartilage. MMPs can be induced via multiple pathways, and inflammatory cytokines such as IL-6 can directly induce the production of MMPs [51]. The present study showed that DHJSD directly inhibited the formation of IL-1β and MMP-13 *in vivo*, thus inducing a protective role for the articular cartilage. Studies have reported that zinc is closely related to the formation of MMPs [52,53]. This implies that when cells synthesize more MMPs in an inflammatory state, more zinc is needed to enter the cells.

**Fig 9:**
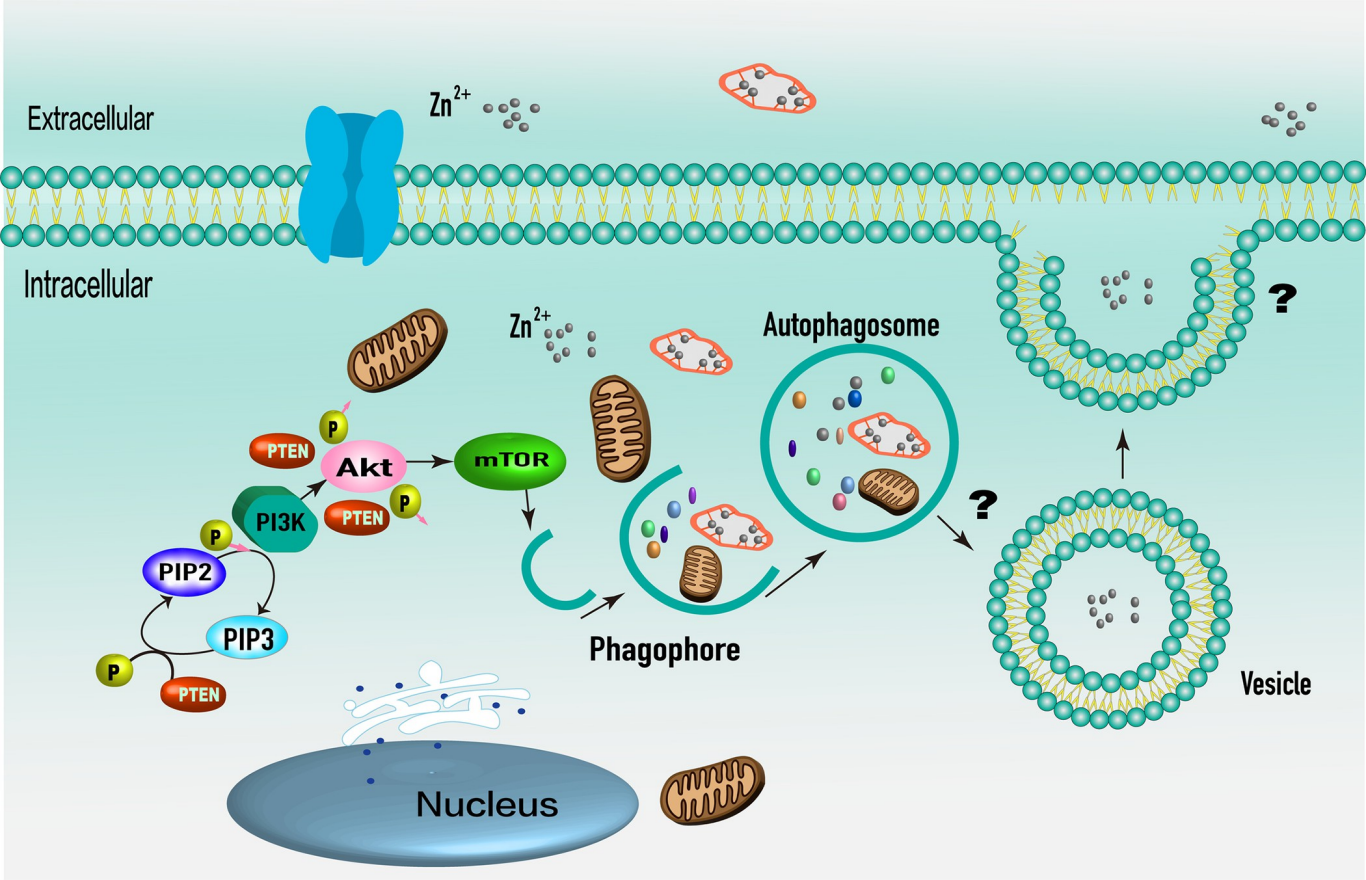
Schematic representation of the mechanism by which DHJSD regulates zinc homeostasis in chondrocytes.

The present study showed that the intracellular zinc level in chondrocytes increased after IL-1β treatment of chondrocytes, and pretreatment with Ds decreased the zinc level in IL-1β-stimulated chondrocytes. These findings indicate that one of the reasons DHJSD may inhibit MMP formation is by downregulating intracellular zinc levels. Thus, DHJSD can enhance the level of autophagy in chondrocytes while also reducing zinc levels in chondrocytes. However, the study could not clarify how zinc is expelled out of cells through autophagy and whether it is related to the cell exocytosis mechanism (**Fig 9**). The findings of this study indicate that the DHJSD maintains intracellular zinc homeostasis by enhancing cellular autophagy; however, further studies are required to clarify this mechanism.

## Conclusion

Histopathological observations verified the improvement in KOA in rats following treatment with DHJSD. Additionally, in the rats with KOA induced using the Hulth method, DHJSD enhanced autophagy by inhibiting the Akt/mTOR signaling pathway by upregulating PTEN protein levels as well as downregulated the levels of proinflammatory cytokines IL-1β and MMP-13. Moreover, *in vitro* findings revealed that DHJSD can enhance the level of autophagy and regulate intracellular zinc homeostasis through the PTEN/Akt/mTOR signaling pathway. Thus, DHJSD has anti-OA and cartilage protective effects in the treatment of KOA both *in vivo* and *in vitro*.

## Supporting information

S1 Fig Positive and negative ion chromatograms. (PDF)

S1 Raw image (PDF)

S2 Raw image (PDF)

## Abbreviations

Akt: V-akt murine thymoma viral oncogene homolog
Bax: BCL2-associated X
Bcl-2: B-cell lymphoma-2
DHJSD: Duhuo Jisheng Decoction
Ds: DHJSD-serum
HD: high-dose
IL-1β: interleukin-1β
ICP-MS/MS: inductively coupled plasma mass spectrometry
KOA: knee osteoarthritis
LC-MS/MS: liquid chromatography tandem mass spectrometry
LC3: microtubule-associated proteins 1A/1B light chain 3
LD: low-dose
MD: medium-dose
mTOR: mammalian target of rapamycin
MMP-13: matrix metallopeptidase 13
OA: osteoarthritis
RT-PCR: real-time PCR
PTEN: phosphatase and tensin homolog
PI3K: phosphoinositide 3 kinase
p-Akt: phosphorylated Akt
p-mTOR: phosphorylated mTOR

## References

[1] Sun X, Zhen X, Hu X, Li Y, Gu S, Gu Y, et al. Osteoarthritis in the Middle-Aged and Elderly in China: Prevalence and Influencing Factors. Int J Environ Res Public Health. 2019;16(23). 10.3390/ijerph16234701 PMC6926632 31779104

[2] Yingying Z, Xudong L, Jiajuan Y, Li M, Yuewei C. The prevalence of osteoarthritis in Chinese aged 40 and over: a meta-analysis. CHINESE JOURNAL OF EVIDENCE-BASED MEDICINE. 2021;21(4): 407–414.

[3] Postler AE, Lützner C, Goronzy J, Lange T, Deckert S, Günther KP, et al. When are patients with osteoarthritis referred for surgery? Best Pract Res Clin Rheumatol. 2023;101835. doi: 10.1016/j.berh.2023.101835 37263807

[4] Gong J, Li Q, Wei M, Xue L, Liu Y, Gao J, et al. Effect of Tongluozhitong Prescription-Assisted Intra-Articular Injection of Sodium Hyaluronate on VAS Score and Knee Lysholm Score in Patients with Knee Osteoarthritis. Evid Based Complement Alternat Med. 2021;20213210494. doi: 10.1155/2021/3210494 34745278 PMC8568548

[5] Ramachandran S, Salkar M, Bentley JP, Eriator I, Yang Y. Patterns of Long-Term Prescription Opioid Use Among Older Adults in the United States: A Study of Medicare Administrative Claims Data. Pain Physician. 2021;24(1): 31–40. doi: 10.1016/j.jpain.2014.05.009 33400426 PMC7789048

[6] Vo NX, Le NNH, Chu TDP, Pham HL, Dinh KXA, Che UTT, et al. Effectiveness and Safety of Glucosamine in Osteoarthritis: A Systematic Review. Pharmacy (Basel). 2023;11(4). doi: 10.3390/pharmacy11040117 37489348 PMC10366893

[7] Honvo G, Reginster JY, Rabenda V, Geerinck A, Mkinsi O, Charles A, et al. Safety of Symptomatic Slow-Acting Drugs for Osteoarthritis: Outcomes of a Systematic Review and Meta-Analysis. Drugs Aging. 2019;36(Suppl 1): 65–99. doi: 10.1007/s40266-019-00662-z 31073924 PMC6509099

[8] Shrestha R, Tamrakar S, Koju P, Maharjan S, Malla S, Shakya Shrestha S. Investigating the Efficacy and Safety of Diacerein in the Management of Knee Osteoarthritis with reference to its conventional management. J Nepal Health Res Counc. 2023;20(4): 942–946. doi: 10.33314/jnhrc.v20i4.4323 37489681

[9] Liu ZC, Wang ZL, Huang CY, Fu ZJ, Liu Y, Wei ZC, et al. Duhuo Jisheng Decoction inhibits SDF-1-induced inflammation and matrix degradation in human degenerative nucleus pulposus cells in vitro through the CXCR4/NF-κB pathway. Acta Pharmacol Sin. 2018;39(6): 912–922. 29795361 10.1038/aps.2018.36 PMC6256264

[10] Guo D, Cheng K, Song C, Liu F, Cai W, Chen J, et al. Mechanisms of inhibition of nucleus pulposus cells pyroptosis through SDF1/CXCR4-NFkB-NLRP3 axis in the treatment of intervertebral disc degeneration by Duhuo Jisheng Decoction. Int Immunopharmacol. 2023;124(Pt A): 110844. doi: 10.1016/j.intimp.2023.110844 37647678

[11] Zhou D, Song C, Mei Y, Cheng K, Liu F, Cai W, et al. A review of Duhuo Jisheng decoction mechanisms in intervertebral disc degeneration in vitro and animal studies. J Orthop Surg Res. 2023;18(1): 436. doi: 10.1186/s13018-023-03869-4 37322524 PMC10273736

[12] Song C, Chen R, Cheng K, Zhou D, Mei Y, Yan J, et al. Exploring the pharmacological mechanism of Duhuo Jisheng Decoction in treating intervertebral disc degeneration based on network pharmacology. Medicine (Baltimore). 2023;102(22): e33917. doi: 10.1097/MD.0000000000033917 37266623 PMC10238016

[13] Gu YT, Chen J, Meng ZL, Ge WY, Bian YY, Cheng SW, et al. Research progress on osteoarthritis treatment mechanisms. Biomed Pharmacother. 2017;931246–1252. doi: 10.1016/j.biopha.2017.07.034 28738541

[14] Wang X, Ning Y, Yang L, Yu F, Guo X. Zinc: the Other Suspected Environmental Factor in Kashin-Beck Disease in Addition to Selenium. Biol Trace Elem Res. 2017;179(2): 178–184. doi: 10.1007/s12011-017-0964-8 28224461

[15] Kim JH, Jeon J, Shin M, Won Y, Lee M, Kwak JS, et al. Regulation of the catabolic cascade in osteoarthritis by the zinc-ZIP8-MTF1 axis. Cell. 2014;156(4): 730–743. doi: 10.1016/j.cell.2014.01.007 24529376

[16] Liuzzi JP, Pazos R. Interplay Between Autophagy and Zinc. J Trace Elem Med Biol. 2020;62126636. doi: 10.1016/j.jtemb.2020.126636 32957075

[17] Yu Z, Yu Z, Chen Z, Yang L, Ma M, Lu S, et al. Zinc chelator TPEN induces pancreatic cancer cell death through causing oxidative stress and inhibiting cell autophagy. J Cell Physiol. 2019;234(11): 20648–20661. doi: 10.1002/jcp.28670 31054150

[18] Shinozaki D, Merkulova EA, Naya L, Horie T, Kanno Y, Seo M, et al. Autophagy Increases Zinc Bioavailability to Avoid Light-Mediated Reactive Oxygen Species Production under Zinc Deficiency. Plant Physiol. 2020;182(3): 1284–1296. doi: 10.1104/pp.19.01522 31941669 PMC7054869

[19] Wang YZ, Wu ZJ, Zhang HY, Zhu MS. Study on the effect of Duhuo-Jisheng Decoction on knee osteoarthritis based on Zinc/ZIP8 signaling pathway. LiShiZhen Med Res. 2020;31(5): 1057–1060.

[20] Xu Z, Han X, Ou D, Liu T, Li Z, Jiang G, et al. Targeting PI3K/AKT/mTOR-mediated autophagy for tumor therapy. Appl Microbiol Biotechnol. 2020;104(2): 575–587. doi: 10.1007/s00253-019-10257-8 31832711

[21] Harikrishnan H, Jantan I, Haque MA, Kumolosasi E. Anti-Inflammatory Effects of Hypophyllanthin and Niranthin Through Downregulation of NF-κB/MAPKs/PI3K-Akt Signaling Pathways. Inflammation. 2018;41(3): 984–995. 29427163 10.1007/s10753-018-0752-4

[22] Turco E, Witt M, Abert C, Bock Bierbaum T, Su MY, Trapannone R, et al. How RB1CC1/FIP200 claws its way to autophagic engulfment of SQSTM1/p62-ubiquitin condensates. Autophagy. 2019;15(8): 1475–1477. doi: 10.1080/15548627.2019.1615306 31066340 PMC6613900

[23] Pinto AP, Da Rocha AL, Marafon BB, Rovina RL, Muñoz VR, Da Silva LECM, et al. Impact of Different Physical Exercises on the Expression of Autophagy Markers in Mice. Int J Mol Sci. 2021;22(5): 2635. doi: 10.3390/ijms22052635 33807902 PMC7962017

[24] Xu W, Yang Z, Xie C, Zhu Y, Shu X, Zhang Z, et al. PTEN lipid phosphatase inactivation links the hippo and PI3K/Akt pathways to induce gastric tumorigenesis. J Exp Clin Cancer Res. 2018;37(1): 198. doi: 10.1186/s13046-018-0795-2 30134988 PMC6104022

[25] Bu L, Wang H, Pan JA, Chen L, Xing F, Wu J, et al. PTEN suppresses tumorigenesis by directly dephosphorylating Akt. Signal Transduct Target Ther. 2021;6(1): 262. doi: 10.1038/s41392-021-00571-x 34248141 PMC8273154

[26] Aquila S, Santoro M, Caputo A, Panno ML, Pezzi V, De Amicis F. The Tumor Suppressor PTEN as Molecular Switch Node Regulating Cell Metabolism and Autophagy: Implications in Immune System and Tumor Microenvironment. Cells. 2020;9(7): 1725. doi: 10.3390/cells9071725 32708484 PMC7408239

[27] Malaney P, Uversky VN, Davé V. PTEN proteoforms in biology and disease. Cell Mol Life Sci. 2017;74(15): 2783–2794. doi: 10.1007/s00018-017-2500-6 28289760 PMC11107534

[28] Wang L, Lu G, Shen HM. The Long and the Short of PTEN in the Regulation of Mitophagy. Front Cell Dev Biol. 2020;8299. doi: 10.3389/fcell.2020.00299 32478067 PMC7237741

[29] Haddadi N, Lin Y, Travis G, Simpson AM, Nassif NT, Mcgowan EM. PTEN/PTENP1: ’Regulating the regulator of RTK-dependent PI3K/Akt signalling’, new targets for cancer therapy. Mol Cancer. 2018;17(1): 37. doi: 10.1186/s12943-018-0803-3 29455665 PMC5817727

[30] Bi X, Li T, Li M, Xiang S, Li J, Ling B, et al. A New Method to Develop the Primate Model of Knee Osteoarthritis With Focal Cartilage Defect. Front Bioeng Biotechnol. 2021;9727643. doi: 10.3389/fbioe.2021.727643 34805105 PMC8599286

[31] Tanvir EM, Whitfield KM, Ng JC, Shaw PN. Development and Validation of an ICP-MS Method and Its Application to Determine Multiple Trace Elements in Small Volumes of Whole Blood and Plasma. J Anal Toxicol. 2021;44(9): 1036–1046. doi: 10.1093/jat/bkaa033 32232355

[32] Laur N, Kinscherf R, Pomytkin K, Kaiser L, Knes O, Deigner HP. ICP-MS trace element analysis in serum and whole blood. PLoS One. 2020;15(5): e0233357. doi: 10.1371/journal.pone.0233357 32433650 PMC7239469

[33] Zheng S, Zhou B, Yang L, Hou A, Zhang J, Yu H, et al. System pharmacology analysis to decipher the effect and mechanism of active ingredients combination from Duhuo Jisheng decoction on osteoarthritis in rats. J Ethnopharmacol. 2023;315116679. doi: 10.1016/j.jep.2023.116679 37257711

[34] Xin P, Xu X, Zhang H, Hu Y, Deng C, Sun S, et al. Mechanism investigation of Duhuo Jisheng pill against rheumatoid arthritis based on a strategy for the integration of network pharmacology, molecular docking and in vivo experimental verification. Pharm Biol. 2023;61(1): 1431–1445. 37674371 10.1080/13880209.2023.2252854 PMC10486301

[35] Zhang Y, Cao L, Du R, Tian F, Li X, Yuan Y, et al. MiR-31 improves spinal cord injury in mice by promoting the migration of bone marrow mesenchymal stem cells. PLoS One. 2022;17(9): e0272499. doi: 10.1371/journal.pone.0272499 36067193 PMC9447891

[36] Leone F, Cataldo R, Mohamed SSY, Manna L, Banchero M, Ronchetti S, et al. Nanostructured ZnO as Multifunctional Carrier for a Green Antibacterial Drug Delivery System-A Feasibility Study. Nanomaterials (Basel). 2019;9(3). 10.3390/nano9030407 PMC6473990 30862002

[37] Ikebuchi Y, Aoki S, Honma M, Hayashi M, Sugamori Y, Khan M, et al. Coupling of bone resorption and formation by RANKL reverse signalling. Nature. 2018;561(7722): 195–200. doi: 10.1038/s41586-018-0482-7 30185903

[38] D’arcy Y, Mantyh P, Yaksh T, Donevan S, Hall J, Sadrarhami M, et al. Treating osteoarthritis pain: mechanisms of action of acetaminophen, nonsteroidal anti-inflammatory drugs, opioids, and nerve growth factor antibodies. Postgrad Med. 2021;133(8): 879–894. doi: 10.1080/00325481.2021.1949199 34252357

[39] Bruno R dC, Pereira TV, Saadat P, Rudnicki M, Iskander SM, Bodmer NS, et al. Effectiveness and safety of non-steroidal anti-inflammatory drugs and opioid treatment for knee and hip osteoarthritis: network meta-analysis. BMJ (Clinical Research ed). 2021;375n2321. 10.1136/bmj.n2321 PMC8506236 34642179

[40] Szeto CC, Sugano K, Wang JG, Fujimoto K, Whittle S, Modi GK, et al. Non-steroidal anti-inflammatory drug (NSAID) therapy in patients with hypertension, cardiovascular, renal or gastrointestinal comorbidities: joint APAGE/APLAR/APSDE/APSH/APSN/PoA recommendations. Gut. 2020;69(4): 617–629. doi: 10.1136/gutjnl-2019-319300 31937550

[41] Xia H, Cao D, Yang F, Yang W, Li W, Liu P, et al. Jiawei Yanghe decoction ameliorates cartilage degradation in vitro and vivo via Wnt/β-catenin signaling pathway. Biomed Pharmacother. 2020;122109708. 10.1016/j.biopha.2019.109708 31918279

[42] Zhang SL, Zhang KS, Wang JF, Wang YC, Zhang H, Tang C, et al. Corresponding Changes of Autophagy-Related Genes and Proteins in Different Stages of Knee Osteoarthritis: An Animal Model Study. Orthop Surg. 2022;14(3): 595–604. doi: 10.1111/os.13057 35088942 PMC8927001

[43] Hou SM, Chen PC, Lin CM, Fang ML, Chi MC, Liu JF. CXCL1 contributes to IL-6 expression in osteoarthritis and rheumatoid arthritis synovial fibroblasts by CXCR2, c-Raf, MAPK, and AP-1 pathway. Arthritis Res Ther. 2020;22(1): 251. doi: 10.1186/s13075-020-02331-8 33087182 PMC7580030

[44] Liu L, Xu L, Wang S, Wang L, Wang X, Xu H, et al. Confirmation of inhibitingTLR4/MyD88/NF-κB Signalling Pathway by Duhuo Jisheng Decoction on Osteoarthritis: A Network Pharmacology Approach-Integrated Experimental Study. Front Pharmacol. 2021;12784822. 10.3389/fphar.2021.784822 PMC8818874 35140604

[45] Sorice M. Crosstalk of Autophagy and Apoptosis. Cells. 2022;11(9): 1479. doi: 10.3390/cells11091479 35563785 PMC9102887

[46] Das S, Shukla N, Singh SS, Kushwaha S, Shrivastava R. Mechanism of interaction between autophagy and apoptosis in cancer. Apoptosis. 2021;26(9–10): 512–533. doi: 10.1007/s10495-021-01687-9 34510317

[47] Sharma A, Almasan A. Autophagy and PTEN in DNA damage-induced senescence. Adv Cancer Res. 2021;150249–284. doi: 10.1016/bs.acr.2021.01.006 33858598

[48] Mehana ESE, Khafaga AF, El-Blehi SS. The role of matrix metalloproteinases in osteoarthritis pathogenesis: An updated review. Life Sci. 2019;234116786. doi: 10.1016/j.lfs.2019.116786 31445934

[49] Liang Y, Xu X, Xu L, Iqbal Z, Ouyang K, Zhang H, et al. Chondrocyte-specific genomic editing enabled by hybrid exosomes for osteoarthritis treatment. Theranostics. 2022;12(11): 4866–4878. doi: 10.7150/thno.69368 35836795 PMC9274754

[50] Hu Q, Ecker M. Overview of MMP-13 as a Promising Target for the Treatment of Osteoarthritis. Int J Mol Sci. 2021;22(4): 1724. 33572320 10.3390/ijms22041742 PMC7916132

[51] Tabeian H, Betti BF, Dos Santos Cirqueira C, De Vries TJ, Lobbezoo F, Ter Linde AV, et al. IL-1β Damages Fibrocartilage and Upregulates MMP-13 Expression in Fibrochondrocytes in the Condyle of the Temporomandibular Joint. Int J Mol Sci. 2019;20(9): 2260. 31067826 10.3390/ijms20092260 PMC6539937

[52] Kim IS, Yang WS, Kim CH. Physiological Properties, Functions, and Trends in the Matrix Metalloproteinase Inhibitors in Inflammation-Mediated Human Diseases. Curr Med Chem. 2023;30(18): 2075–2112. doi: 10.2174/0929867329666220823112731 36017851

[53] Nosrati R, Kheirouri S, Ghodsi R, Ojaghi H. The effects of zinc treatment on matrix metalloproteinases: A systematic review. J Trace Elem Med Biol. 2019;56107–115. doi: 10.1016/j.jtemb.2019.08.001 31442948

