# Correction: Duhuo Jisheng Decoction regulates intracellular zinc homeostasis by enhancing autophagy via PTEN/Akt/mTOR pathway to improve knee cartilage degeneration

**DOI:** 10.1371/journal.pone.0306763

**Published:** 2024-07-03

**Authors:** Ye-Hui Wang, Yi Zhou, Xiang Gao, Sheng Sun, Yi-Zhou Xie, You-Peng Hu, Yang Fu, Xiao-Hong Fan, Quan Xie

The affiliation for the ninth author is incorrect. Quan Xie is not affiliated with #2 but with #1: Hospital of Chengdu University of Traditional Chinese Medicine, Chengdu, Sichuan, China
