# Supplementary figures and images for: Duhuo Jisheng Decoction regulates intracellular zinc homeostasis by enhancing autophagy via PTEN/Akt/mTOR pathway to improve knee cartilage degeneration
Source: PLoS One. 2024 Jan 2;19(1):e0290925. doi: 10.1371/journal.pone.0290925 (PMC10760926; doi:10.1371/journal.pone.0290925)

## Positive and negative ion chromatograms

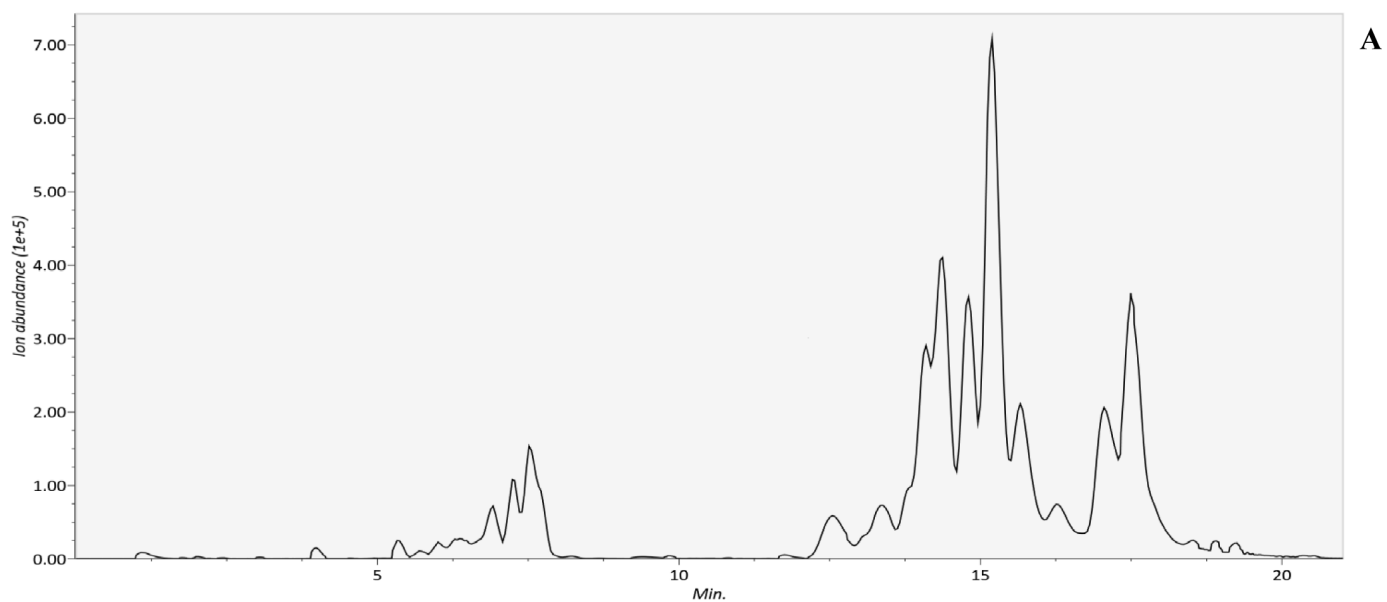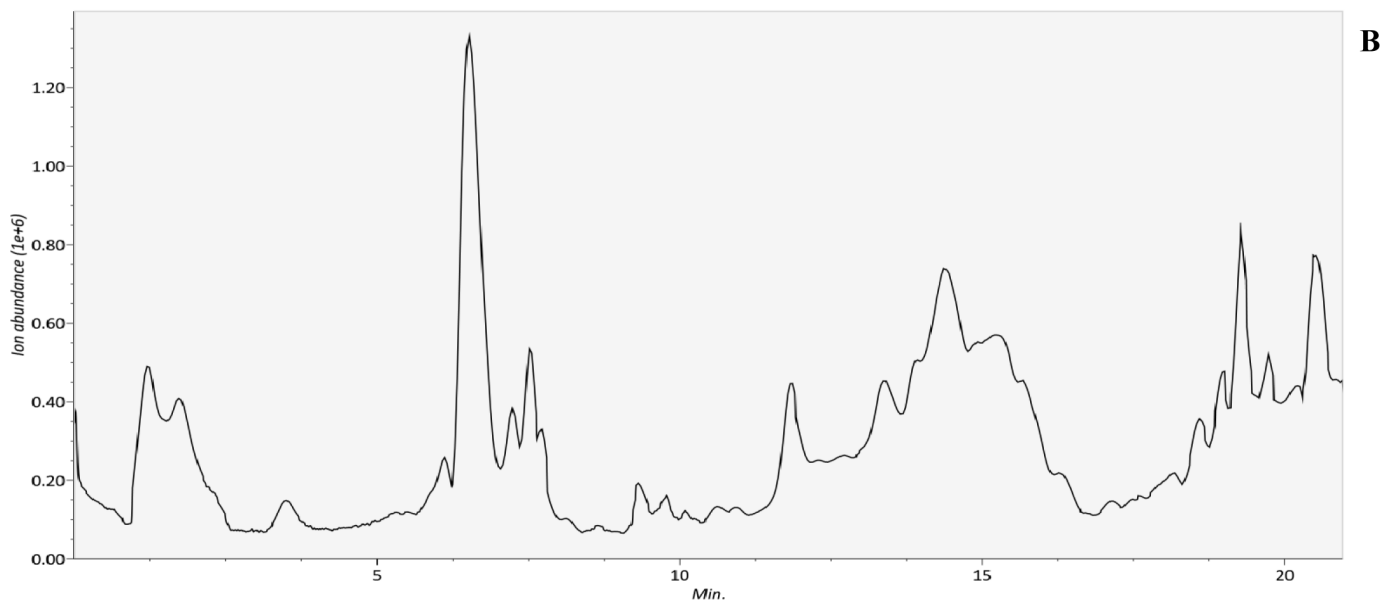

Supplement: S1 Fig — (PDF) [file pone.0290925.s001.pdf]
