# Supplementary material for: Duhuo Jisheng Decoction regulates intracellular zinc homeostasis by enhancing autophagy via PTEN/Akt/mTOR pathway to improve knee cartilage degeneration
Source: PLoS One. 2024 Jan 2;19(1):e0290925. doi: 10.1371/journal.pone.0290925 (PMC10760926; doi:10.1371/journal.pone.0290925)

vivo For Figs 4-5

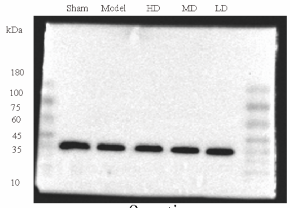

β-actin

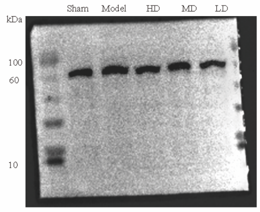

Akt

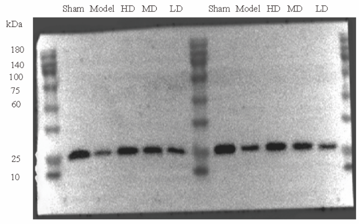

Bcl-2

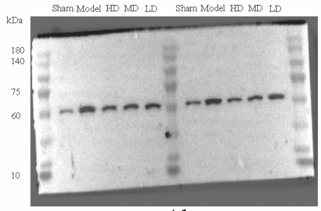

p-Akt

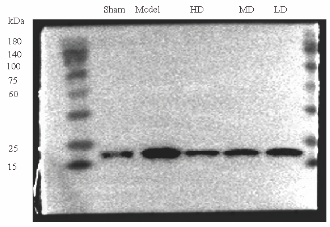

Bax

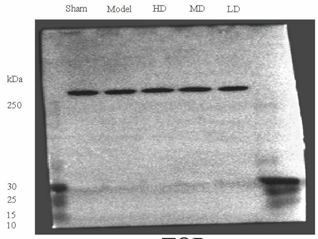

mTOR

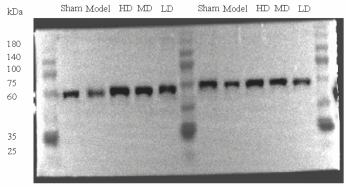

Beclin-1

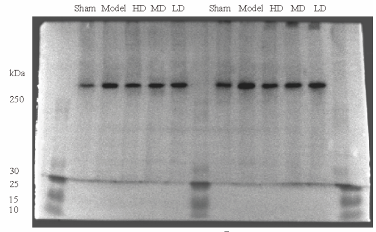

p-mTOR

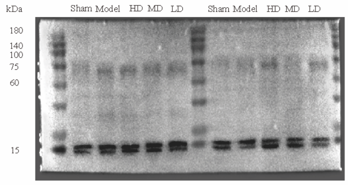

LC3

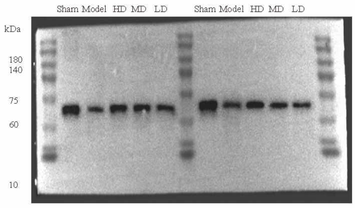

PTEN

Tianneng GIS chassis control software V2.0 was used to obtain the original images,
and Image lab (6.1) was used to merge the images. The images were typesetted using Adobe Illustrator (27.0).

vitro For Figs 7-8

Tianneng GIS chassis control software V2.0
was used to obtain the original images,
and Image lab (6.1) was used to merge the images.
The images were typesetted using Adobe Illustrator (27.0).

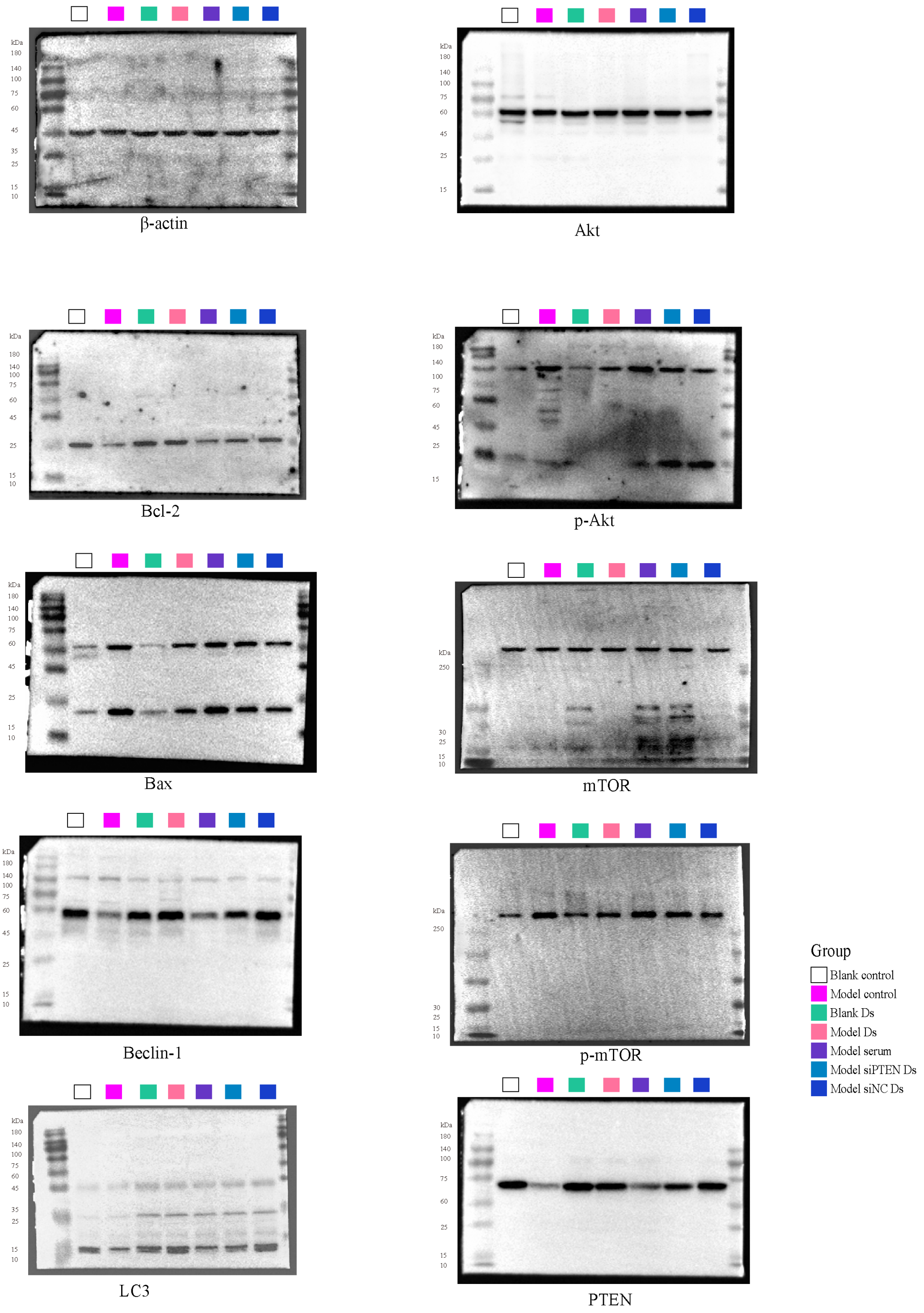

Supplement: S2 Raw image — (PDF) [file pone.0290925.s003.pdf]
